# On the Radical Cure of Varices, Deduced from the Proximate Cause

**Published:** 1847-11

**Authors:** Tommaso Rima

**Affiliations:** Ordinary Member of the Athenæum, Senior Surgeon to the Civil Provincial Hospital of Venice, formerly Chief Surgeon of the Navy, Clinical Professor, and Surgeon in Chief to the Military Hospital, of the late Kingdom of Italy


*On the radical cure of Varices, deduced from the proximate cause. Explained and demonstrated in Two Memoirs, by* Dr. Tommaso Rima; Ordinary Member of the Athenæum, Senior Surgeon to the Civil Provincial Hospital of Venice, formerly Chief Surgeon of the Navy, Clinical Professor, and Surgeon in Chief to the Military Hospital, of the late Kingdom of Italy. Rewarded with the Golden Medal of S. M. I. R., Ferdinand First, from the Royal Institute, for the invention of permanent and moveable swimming baths, &c., &c., &c. *Second Edition, with an appendix by the Author. Translated from the Italian*, by James Bryan, M. D., Lecturer on Surgery, &c., &c.

*On Varicocele.*

In the classification of the innumerable disorders to which the human organism is subject, some are alterations of tissue, which sooner or later according to the importance of the part attacked, tend to the destruction of life; others are so circumscribed in their effects, that they occasion only a slight alteration in the function of the organ or viscera where they are seated. Thus they sometimes disappear spontaneously, instead of increasing with advancing years. But some of these occasionally remain within certain limits, and produce an amount of suffering disproportioned to the actual extent of the organic lesion. Of this number is Varicocele. This consists, as is well known, in a morbid enlargement of the veins which convey the blood from the testicle to the general circulation, coursing, enclosed and covered by the scrotum, along the spermatic cord, commonly in an irregular serpentine matter.

It will not be in place here to speak of the essential character of this disease, of the degree of alteration which the membranous tissues of the spermatic veins undergo, of the mode of distinguishing it from hernia of the omentum, or of other diseases with which it may be confounded. We will merely state, to fix it in its place, not to verify the disease, as all writers have asserted, one characteristic sign of Varicocele, which will distinguish it from omental hernia: it is, that the former developes itself from the testicle towards the abdominal ring, while the latter, or epiplocele, descends from the inguinal canal to the scrotum. A theory which is well proved by physiological and pathological laws, is not always established by a clinic, at the bedside of the patient. There is no doubt but that an epiplocele must descend from above, and pass the inguinal canal into the scrotum. But we cannot so certainly say that a varicocele must pass from below upwards, from the testicle to the ring. If we admit (for the moment) a new supposition, that the blood in varicocele may descend from the vena cava and renal veins, and oppose that which naturally ascends from the testicle to the spermatic veins. This symptom would cease to be pathognomic, and the obscure would become diagnostic. We will return to the argument when it becomes necessary to prove that such a supposition may be demonstrated by facts.

In the majority of cases, varicocele remains latent and innocuous. It is, for the most part, by mere accident that the most careful discover it under the fingers. It generally appears as a group of worms, covered by the integuments of the scrotum, merely inducing a sense of weight in the testicle, a dull sense of stretching in the region of the kidney, and this not constantly. The patient, half mechanically, supports his scrotum for relief. With this precaution he walks, rides, dances and undergoes any other gymnastic exercise without pain.

Our predecessors limited their prescriptions to a suspensory bandage, local astringents, bathing and cold water. The cure was merely palliative. We do not speak of certain empirical ministers of Hygeia, who applied various pharmaceutical preparations, whether sanctioned by experience and reason, or not, to all diseases.

All surgeons, who are not entirely unknown, will occasionally have patients present themselves to be relieved of varicocele. Some apply to us to relieve them of a varicocele, which is neither voluminous, nor incompatible with the uses of life. Here, it will not be difficult to convince the patient, that greater harm will result from the operation, than the benefit which can be hoped for.

Though it is true that this is the ordinary course of the disease, yet it is very certain that, in some rare cases, pains and other symptoms arise where it is permitted to push the knife beyond the limits which antiquated scholastic precepts have in vain imposed on an illimitable art. The following is a case in point:—Antonio Morbiato, of Venice, writer under the royal pretorship, in the parish of Cadore, came to this country in July, 1833, to place himself under my care for a varicocele in the left testicle—a more frequent seat of this disease than the right.

He had arrived at the thirty-second year of his age, and had no appearance of want of health. For ten years past the varicocele had been so decided as to exempt him from military duty. But it continued so mild as not to interfere with his running over the Alpine rocks of the Cadorino in the chase, which was his favourite amusement. Not until November of 1822, (writes the patient) did the first painful sensation pass along a varicose and tortuous vein, which had ascended towards the testicle, swelled so visibly as to exempt me from military conscription, for the six months previous. On consulting a physician he recommended to me the use of cold bathing in water and vinegar, with the occasional use of the suspensory bandage. He did not, however, give up his favourite exercise, the chase, or restrain himself from indulgence in that which his vigorous age inclined him to. Fortunately, notwithstanding this violent exercise, after a time the painful sensation left him so completely, that he was enabled for seven years to follow his Alpine course.

The slight pain was confined entirely to the enlarged vein, whose swelling increased by exercise, and particularly during four or five days in the spring of the year. With this slight variation, the life of our patient continued up to 1833. It must be recollected that in March the disease increased continually, but with little pain. The means first resorted to, with repeated leeching, were insufficient to impede its progress, so that in the middle of July he writes, his life had become burdensome, since he was constantly confined to his bed, full of pain and fit for nothing.

The cure was not completed on the 20th of July. The infusion of digitalis and guaiacum, locally applied, diminished for a time his sufferings, but was not sufficient to induce me to expect a cure, without the repetition of leeches. Scarcely had he abandoned the horizontal position, ere his pains recommenced. It appeared, and was a fact, that the blood, in opposition to the natural laws of the circulation, was not moved by the abundance of that passing through the spermatic veins of the testicle, but condensed into one column, in a manner to compress materially by its mass the spermatic nerve. Hence came the unpleasant sensation about the kidney, and not by the other filaments of the mesenteric plexus of the great sympathetic and of the lumbar nerves. To the advantage of the horizontal position, he added, temporarily, that of cold, and of the constant application of guaiacum. This, operating as a mechanical astringent, must necessarily corrugate the vascular tunic on the slow moving column of blood, diminishing its volume and the capacity of the tunic. Such an impression of cold diminishes the chief pressure on the nerves. The sensation is reduced by the action of this agent to an unpleasant sensation of weight and stretching. Changing the supine position of the body exacerbates the suffering. To the most painful, excruciating, and insufferable physical sufferings of the patient, are added severe mental distress; hence, he writes that he is tired of his existence. He is in such a state that he demands succour and efficacious relief. The destruction of the testicle offers, according to the usual practice, the only hope of a cure, but with a painful sacrifice.

The patient will not object, nay, he will implore the operation. But the surgeon of the nineteenth century should not perform it. The radical cure of varices of the leg has been practiced by us in different ways, and repeatedly during the last twenty-six years. In spite of incision, ligature, and the excision of a portion of the saphena, though the result has not corresponded entirely in all, varying in different cases, yet it is proved that the venous circulation has received no harm. This reflection has induced the idea, that as in the great saphena, so in the spermatic veins, no harm can occur. It is true, that these being isolated by a very slight adhesion to the cellular tissue of the scrotum, do not present the most favourable combination in connection, with an infinite number of smaller ramifications. We might fear that if varicocele were formed by a morbid alteration of both the spermatic veins, with the excision of a portion of these the blood of the testicle could not be conducted into the current of the general circulation. As a consequence we might have the disorganization of the testicle. We must be prepared for this accident. But why be not willing to trust the hazard of the operation? The following reflections have induced me to attempt it.

Scarpa has demonstrated, in a masterly manner, in the arterial apparatus, the system of compensation which nature adopts when a vessel has been obliterated, either artificially or by disease. There is reason to believe that, to a certain extent, this physiological and anatomical law is also active in the venous system. If the rule be correct, that greater evils follow the lesion of an artery than that of a vein, it will apply with much more force to operations on the spermatic vessels. There are two veins devoted to this function, besides the artery. This carries the blood for the nutrition of the testicle, and also the principle of which is composed, and from which is elaborated, the first element of life, the semen. The veins have but a secondary office, that of carrying again into the current of the general circulation the residuary blood, in order to be deprived of its carbon and to reacquire its lost oxygen. The venous blood (to avail myself of a trivial idea, but one adapted to my purposes) is the serous portion of the milk, from which is extracted the caseum and buttery portion. The spermatic artery runs in the central part of the cord, while the veins course in a serpentine manner on its external surface. A portion of a vein may therefore be cut without wounding the artery. In cures by injection or ligation of the spermatic vessels, those of the scrotum and dartos coming from the femoral and obturator arteries, join with those of the involucre of the testicle to support its vitality. We may not perhaps expect the secretion of the semen, on account of the smallness of these external vessels, or perhaps the blood of these vessels does not contain the prolific principles which form the elements of this fluid. In our case, of treatment by incision of the veins, where their action is simply secondary, and restricted to carrying the blood, already deprived of its principal elements, these vessels may, on account of their tenuity, carry on the function in substitution of the spermatic veins, when the latter are entirely destroyed. As the two veins correspond to one artery, each of which is larger than the artery itself, we must hence admit, that the venous circulation is more certain than the arterial. The compensation to the venous system may be the lymphatic vessels. Besides performing their own functions, these vessels may carry venous blood. From the experiments of Lippi, and the controversy of Rossi, we are informed that there exist direct communications between some of the venous and lymphatic vessels.

The operator and patient being agreed, it was decided to commence with the incision of the varicose veins alone, and to cut successively the artery and vas deferens, should the sacrifice of the testicle become indispensable.

Such an operation, sanctioned by a distinguished practitioner of this city, was in fact directed on the seventh of August, 1833, with the assistance of Dr. Trevesini, royal fiscal physician. A transverse portion of the integuments was elevated, from which a perpendicular incision about two inches in extent was made. Raising with a pair of forceps the fascia of the varicose veins, three incisions were made with a pair of scissors, for the purpose of exposing the veins which surround the vas deferens. The third incision was much the most painful, corresponding to the track of the vein, whose great pain induced the patient at any risk to submit to the operation. There was no necessity for ligatures on the vessels; the hæmorrhage being purely venous, ceased spontaneously. The wound was dressed with a linen cloth tempered with oil and lint, spread with a refrigerating ointment. No phenomena worthy of notice were developed, the pulse scarcely indicating fever. Forty-eight hours afterwards, the wound was dressed with little or no inconvenience to the patient. This could not be said three hours afterwards. Severe pains attacked the patient, ascending from the lower part of the wound to the region of the kidney, which were increased by the least motion of the body; so that the patient was reduced to a tetanic immobility imploring assistance. The pulse was depressed and jerking, subsultus tendinum, extremities cold; eyes sunk; hippocratic countenance, with cold sweats, were the symptoms presented. The calm through which he had passed for fifty-two hours, after the operation, did not indicate a scene so alarming. This sudden and violent attack was a speaking proof, that the deleterious influence of some morbid agent was acting upon the nervous system. Could it be the atmospheric air admitted for an instant during the last dressing? Assafœtida with morphia internally; an embrocation of oil and morphia to the wound, with the application of guaiacum to the groin and to the renal region, were the means resorted to, during two days of great suffering; also, a blood letting with the application of leeches to lumbar region, as soon as vital reaction would permit. All the so-called nervous symptoms having subsided, about the third day, general inflammation attacked the wound and vicinity. Moderated by the abstraction of blood, digitalis and local applications, intended to reduce vascular action, a laudable and abundant suppuration put an end to the painful scene of this morbid process. The testicle which had remained safe during the storm, became also, by the affusion from the wounded part, inflamed, and was the seat of a collection of fœtid pus, in the tunica vaginalis and albuginea, which discharged spontaneouslythrough the wound. From this time the ulcer progressed regularly, and the cicatrix was healed early in September. Sig. Morbiato returned to the duties of his office, and soon after to his usual habits.

I have now finished the historical part of the operation. It remains to add certain reflexions, as well on practical surgery, as on the special pathology of the veins. When the intolerable pains of our patient induced him to submit to the operation, we were not aware that others had performed it. The common practice, supported particulary by the authority of Boyer, has been the demolition of the testicle. In the history of medicine we learn that various varicose tumours have been extirpated by good surgeons. Petit has excised two, one of which he calls lymphatic sanguineous varicocele. The other, which was much less, he excised from the cord. A third was eradicated by my friend and colleague, Dr. Cumeno, of Trieste, from a French military surgeon, who does not speak of simple varices of the spermatic veins, but of varicose tumours. These tumours may form independently of the veins. We know that cancer from its nature produces varicose veins, and there is cancer of the varices. From the small vessels of the scrotal membrane, one of those inorganic fungous productions may arise, which may have but a superficial, or perhaps no attachment to the spermatic veins. If this were known it might be extirpated, leaving one of the two untouched. In order then to arrange the facts and suppositions above written, we have proved--

1st. That varicocele, under ordinary circumstances, is a disease of little inconvenience, yet occasionally it becomes so painful as to render it intolerable, and to demand the assistance of the surgeon.

2d. That varicocele may be radically cured by the excision of the varicose mass, without the destruction of the testicle, as was the practice of our predecessors.

3d. That the serious consideration of surgeons should be directed to the theory of varices, to know whether generally, or in some solitary cases, the disease takes place, or is maintained by the blood which flows back from the great veins. This should be suspected by writers on varicocele, as I have sufficiently demonstrated in a letter of December 29th, 1825, speaking of varices of the lower extremities. Whilst I operated on the seventh of August, 1833, Sig. Dufresne published, on the 27th February, of the present year 1836, in the Journal Hebdomadaire, a memoir containing various operative processes for the radical cure of varicocele. He refers to the practice of the ancients, who tied the vein above or below the varicose group; he does not analyse this operative process and give the rule, but merely writes it as a historical fact.

We learn from this memoir that the learned physician, our colleague and friend, Professor Delpech, the unhappy victim of the ingratitude of men, has operated several times, with happy results, by the excision of the varicose spermatic veins. Nevertheless, one is surprised that he adheres to the opinion of Boyer, preferring semi-castration. The result is certainly not less doubtful, and the sacrifice is too painful, especially if the patient be in the bloom of life. He then proceeds to give us the opinions and various processes of Breschet, Velpeau, Friche. They attempt in various ways to obtain their purpose without cutting the integuments of the scrotum, and without exposing the vein. Breschet uses a pair of pincers, which somewhat resembles the compressor of Assalini. The vein and a portion of the integuments of the scrotum being introduced between its two branches, they are pressed together so as to bring the parietes of the vein in contact. The others use a needle to perforate the vein, and carry a thread to act as a seton, allowing it to remain two or more days. Others with a greater probability of success, on a needle which has passed through the integuments of the scrotum, under the vein, twist a thread in the figure of 8 form, or else retain the vein in constant compression between the needle and the thread, as is done in hare-lip.

Several cures have been obtained by these processes. He cites among others as authority, the name of Velpeau. But they seem to us not to unite the cito, tuto et jucunde of Celsus, and should not supersede simple excision. It is certain that this pricking terrifies the patient, nor when accomplished is the pain relieved. By incision the pain is transient, and the operation decisive, and is finished in a few seconds. We cannot say so much of the strangulation of the vein between the needle and thread. This occasions a little pain at first, but the pain must of necessity continue to increase, indeterminately, for several days; nor can we know in anticipation or perhaps in reality, to what extent, and how long a time it will continue and excite inflammation, as we will not have the power of limiting it to the simple grade of adhesion. The excited sensibility of the vein does not remain circumscribed to the point compressed. It extends easily along the vein, and there is danger that phlebitis will extend to the larger trunks, endangering the life of the patient. Nor is the inflammation confined to the vein. The integument and other parts which sympathise with it are likely to participate. It is not uncommon for gangrene to attack the integuments which surround the points compressed, and killed, by the pincers or by the needle and thread.

Resection, on the other hand, induces some loss of blood, which depends upon the operator favoring it or not, so that the inflammation of the wounded part may be circumscribed, which cannot be done with compression. Strangulation of the vein does not give confidence to the patient, who perceives always the uncertainty of the cure, without knowing the extent of suffering. Nor should we be surprised if after three or four days the sufferings of the patient should induce the surgeon to relax the compression, leaving him exposed to all the results of the experiment, which are not only useless in effect, but always pernicious, and may induce grave consequences.

In excision, the patient may be compared to a ship in a troubled sea, the wind having ceased to blow; while compression may be compared to a vessel which leaves the shore, throwing itself into an ocean, soon to suffer all the effects of an inevitable tempest, whose duration or termination canot be surmised.

Should the patient have enough of courage and patience to resign himself to events; uncertain whether adhesion will take place, or how much time must pass ere its accomplishment, well; if not, he will consider the operation a total loss. If it is performed in a place which may be obliterated, we may not obtain cure on account of the anastomosis of the obliterated vessel. If the varicocele is in both veins, the operation must be performed the second time, uncertain still whether compression is applied to the remaining vessel or to that which has been operated upon. Facts, in fine, in the cure obtained by strangulation, have proved, as has been said by Dr. Dufresne, that we always have more or less suppuration. Nor can we guarantee the patient from the danger of secondary hæmorrhage, which, without the degeneration of the wound, cannot take place after excision of the vein. Subjected to an impartial examination, and placed in opposition to the advantages we may hope for, and the dangers we may fear, it appears to us that excision of the varicose veins merits the preference without regard to the choice of the patient.

Clinical teachers, who reason upon and compare the different methods, are authorized by experience, to form a judgment which will serve as a guide to the novice in the noble art of surgery.

